# Supplementary material for: Evidence of Intragenic Recombination in African Horse Sickness Virus
Source: Viruses. 2019 Jul 18;11(7):654. doi: 10.3390/v11070654 (PMC6669442; doi:10.3390/v11070654)
Supplement: Supplementary file 1 [file viruses-11-00654-s001.pdf]

Table S1. Origin, isolate name and passage history of AHSV isolates used for the sequence analyses used in this study.

Serotype	Name	Passage history	Year of isolation	Country of origin
1	AHSV-1_29_62	1H,1A	1962	RSA
1	AHSV-1_22_01	Spl,1S,2BHK	2001	RSA
1	AHSV-1_29_02	Spl, 1S, 1V, 2 BHK	2002	RSA
1	AHSV-1_44_00	Spl,	2000	RSA
1	AHSV-1_107_09	2V	2009	RSA
2	AHSV-2_20_09	2V,1V	2009	RSA
2	AHSV-2_82_61	1H,2S	1961	RSA
2	AHSV-2_40_00	Bld,2S,2BHK	2000	RSA
2	AHSV-2_20_01	Spl,1S	2001	RSA
2	AHSV-2_6_81	Spl,	1981	Zim
2	AHSV-2_81_03	?	2003	?
3	AHSV-3_109_08	1V	2008	RSA
3	AHSV-3_13_63	3S	1963	RSA
3	AHSV-3_DG 25324_14	Bld	2014	RSA
3	AHSV-3_DG 25327_14	Bld	2014	RSA
3	AHSV-3_DG 25423_14	Bld	2014	RSA
3	AHSV-3_14_98	Spl, 3V	1998	RSA
3	AHSV-3_48_01	Spl,	2001	RSA
3	AHSV-3_1_02	Spl, 2S, 1V, 1BHK	2002	RSA
3	AHSV-3_2_89	1S	1989	RSA
4	AHSV-4_68_09	2V	2009	RSA
4	AHSV-4_32_62	Spl	1962	Zim
4	AHSV-4_31_00	Bld,1S,3BHK	2000	RSA
4	AHSV-4_91_00	Spl,1S,4BHK	2000	RSA
4	AHSV-4_19_97	2S,1V	1997	
4	AHSV-4_37_98	3V	1998	
4	AHSV-4_65_00	Bld,1S,2BHK	2000	RSA
4	AHSV-4_97_99	1S,2V	1999	RSA
4	AHSV-4_6_14	Lung	2014	Nam
4	AHSV-4_1927_14	Lung	2014	Nam
4	AHSV-4_64_99	1S, 2V	1999	RSA
4	AHSV-4_90_96	Spl, 1V	1996	RSA
4	AHSV-4_90_01	3S	2001	RSA
5	AHSV-5_30_62	2H,1mb	1962	RSA
5	AHSV-5_35_09	3V	2009	RSA
5	AHSV-5_13_94	1V	1994	
5	AHSV-5_93_00	Bld, 2S, 2 BHK	2000	RSA

5	AHSV-5_42_01	Spl, 2S	2001	RSA
5	AHSV-5_13_99	1S, 2V	1999	RSA
5	AHSV-5_86_99	Liver, 1S, 1V	1999	RSA
5	AHSV-5_86_94	Lung, 2V	1994	RSA
5	AHSV-5_47_86	Bld, 4BHK	1986	RSA
5	AHSV-5_2_96	1V	1996	RSA
6	AHSV-6_63_09	3V	2009	RSA
6	AHSV-6_4_98	2V	1998	RSA
6	AHSV-6_2_75	Bld, 2S, 4 BHK	1975	RSA
6	AHSV-6_6_90	Lung, 3V	1990	RSA
6	AHSV-6_68_00	Spl, 1S, 1 BHK	2000	RSA
6	AHSV-6_19_98	Spl, 3V	1998	RSA
6	AHSV-6_33-1_98	Spl, 2V	1998	RSA
6	AHSV-6_110_00	Spl, 1S, 1V, 1 BHK	2000	RSA
6	AHSV-6_14_01	Spl, 1S, 2 BHK	2001	RSA
6	AHSV-6_28_87		1987	
6	AHSV-6_26_03	Bld, 1S, 1 BHK	2003	RSA
6	AHSV-6_93_99	1S, 2V	1999	RSA
6	AHSV-6_33_98	Spl, 2V	1998	RSA
7	AHSV-7_89_09	2V	2009	RSA
7	AHSV-7_31_62	1H,1mb	1962	RSA
7	AHSV-7_1955_14		2014	RSA
7	AHSV-7_67_99	1S, 2V	1999	RSA
7	AHSV-7_34_01	Spl, 2S, 1V, 1BHK	2001	RSA
7	AHSV-7_3_99	1S, 1v	1999	RSA
7	AHSV-7_105_99	1S, 2V,	1999	RSA
8	AHSV-8_3_00		2000	
8	AHSV-8_29_00	1S,4BHK	2000	RSA
8	AHSV-8_10_62	1A	1962	Kenya
8	AHSV-8_96_94	Lung, 1V	1994	RSA
8	AHSV-8_21_99	1S, 2V	1999	RSA
8	AHSV-8_2_99	1S, 2V	1999	RSA
8	AHSV-8_48_98	Spl, 3V	1998	RSA
8	AHSV-8_88_99	1S, 2V	1999	RSA
8	AHSV-8_9_98	Bld, 3V	1998	RSA
8	AH**S**V-8_9B_98	Bld, 3V	1998	RSA
8	AHSV-8_17_98	Spl, 3V	1998	RSA
8	AHSV-8_58_81	Lung, 1S	1981	RSA
9	AHSV-9_90_61	3S	1961	Chad
9	AHSV-9_38_09	2V	2009	RSA
9	AHSV-9_1945_14	?	2014	RSA
9	AHSV-9_2033_14	?	2014	Moz
9	AHSV-9_3_14	?	2014	RSA
9	AHSV-9_43_01	Spl,2S,1V,1BHK	2001	RSA
9	AHSV-9_2_03	Spl, 1S, 1V, 1 BHK	2003	RSA
9	AHSV-9_49_02	Spl, 1S, 2 BHK	2002	RSA

9	AHSV-9_22_03	Spl, 3V	2003	RSA
9	AHSV-9_15_02	Spl, 1S, 1V, 1 BHK	2002	RSA
9	AHSV-9_22_03	Lung, 1S, 1 BHK	2003	RSA
9	AHSV-9_6_01	2 BHK	2001	RSA

Origin of sample: Spl = equine spleen; Bld = equine blood; Lung = equine lung. Number of passages in: H = horse; mb = mouse brain; S = suckling mouse brain; V and BHK = Vero and BHK cell culture passages.
